# Assessment of the In Vivo and In Vitro Release of Chemical Compounds from *Vespa velutina*

**DOI:** 10.3390/molecules26226769

**Published:** 2021-11-09

**Authors:** M. Shantal Rodríguez-Flores, Soraia I. Falcão, Olga Escuredo, Luis Queijo, M. Carmen Seijo, Miguel Vilas-Boas

**Affiliations:** 1 Department of Plant Biology and Soil Sciences, Facultad de Ciencias, Campus As Lagoas, University of Vigo, 32004 Ourense, Spain; oescuredo@uvigo.es (O.E.); mcoello@uvigo.es (M.C.S.); 2 Centro de Investigação de Montanha (CIMO), Polytechnic Institute of Bragança, Campus de Santa Apolónia, 5300-253 Bragança, Portugal; sfalcao@ipb.pt (S.I.F.); mvboas@ipb.pt (M.V.-B.); 3 Department of Mechanical Technology, Polytechnic Institute of Bragança, Campus de Santa Apolónia, 5300-253 Bragança, Portugal; lqueijo@ipb.pt

**Keywords:** HS-SPME/GC-MS, nonanal, pentadecane 8-hexyl-, pest, venom apparatus, *Vespa velutina*

## Abstract

*Vespa velutina* has been rapidly expanding throughout Galicia since 2012. It is causing human health risks and well-known losses in the beekeeping sector. Control methods are scarce, unspecific, and ineffective. Semiochemicals are insect-derived chemicals that play a role in communication and they could be used an integrated pest management tool alternative to conventional pesticides. A previous determination of the organic chemical profile should be the first step in the study of these semiochemicals. HS-SPME in living individuals and the sting apparatus extraction followed by GC-MS spectrometry were combined to extract a possible profile of these compounds in 43 hornets from Galicia. The identified compounds were hydrocarbons, ketones, terpenes, and fatty acid, and fatty acid esters. Nonanal aldehyde appeared in important concentrations in living individuals. While pentadecane, 8-hexyl- and ethyl oleate were mainly extracted from the venom apparatus. Ketones 2-nonanone, 2-undecanone and 7-nonen-2-one, 4,8-dimethyl- were identified by both procedures, as was 1,7-Nonadiene, 4,8-dimethyl-. Some compounds were detected for the first time in *V. velutina* such as naphthalene, 1,6-dimethyl-4-(1-methylethyl). The chemical profile by caste was also characterized.

## 1. Introduction

*Vespa velutina nigrithorax* is an invasive alien species (IAS) included in the list of Exotic Species of Concern for the European Union. The yellow-legged hornet was detected for the first time in 2012 in Galicia (northwest the Iberian Peninsula) and currently, more than 20,000 nests are registered each year [1]. The species is characterized by a rapid spreading, causing economic losses, human health risks, and having a great impact on beekeeping since it is a predator of honeybees. Bait-trapping is one of the most used methods so far to mitigate its impact, but the effectiveness is low and non-specific, affecting the biodiversity of the local entomofauna [1]. The employment of specific pheromones or other semiochemicals to attract *V. velutina* is a future challenge for this pest management. Thereby, the characterization of these compounds could be very useful in preventing damages.

Communication is important in a social species such as *V. velutina*. As other social species, this hornet can communicate through chemical signals. Among other recognition patterns [2], chemical signals mediate communication between nestmates [3], colony activity, or some biological functions such as reproduction [4,5,6] and defense [7,8]. These signals can also intervene in the recognition of morphophysiological traits that are likely to be indicators of the caste [5]. There are different organs or sensory channels, such as antennae, receptors for chemical or visual information, and glands or other parts of the body, such as the cuticle, that produce or recognize these chemical signals. Many insects have developed unique mixtures of cuticular hydrocarbon components (CHCs) that provide informative clues [3]. Cuticular hydrocarbons play an important role in chemical communication, performing functions such as sexual pheromones, allelomones, caste and nestmates recognition signals, dominance signals and fertility, chemical mimicry, primer pheromones, task-specific signals, or even clues to hibernation sites [9,10,11].

In terms of defense and alert, there is a diversity of pheromones and other semiochemical compounds that control this behavior. Although the mandibular gland can also generate alarm compounds, *V. velutina* uses sting venom volatiles as alarm pheromones [7]. When the hornet stings, the venom gland releases the alarm pheromone, which prompts other workers to sting in the same area [7,8,12]. The sting of hornets is only found in females most likely because it evolved from an ovipositor. Therefore, it is not surprising that sex pheromones have also been identified from the venom gland [4,6,13]. These compounds are poorly understood, despite being able to control a variety of behavior patterns, depending on the concentration and other conditions. Thereby, an alarm pheromone can induce search, approach, recruitment, alertness, attack, or repulsion, and a sex pheromone can control alertness, flying against the wind, perching, orientation toward an odorant gradient, and copulation.

Headspace solid-phase microextraction (HS-SPME) is one of the main extraction techniques used to capture volatile organic compounds (VOCs). One of the advantages of this technique is the non-destructive sampling, which can work with specimens in vivo. Thereby, it is possible to work with VOCs emitted directly from *V. velutina*. At the same time, the direct extraction of the sting apparatus allows to analyze the compounds secreted by the venom gland.

Chemical compounds released by *V. velutina* are poorly known, however cuticle hydrocarbons [3,10,14], and other alarm or sexual semiochemicals were recently studied [5,6,7,8,15]. Therefore, this study aims to expand this information by using HS-SPME/GC-MS to characterize the chemicals compounds that *V. velutina* emits in vivo and by studying other possible chemicals compounds obtained from the venom gland by an in vitro extraction method.

## 2. Results

### 2.1. VOCs Collected In Vivo by HS-SPME/GC-MS

HS-SPME extraction allowed the identification of eight volatile compounds released from hornets (Table 1).

Among them were aldehydes such as nonanal and decanal, the ketones 2-nonanone; 2-undecanone; 1,7-nonadiene, 4,8-dimethyl-; 7-nonen-2-one, 4,8-dimethyl-, and geranyl acetone. Finally, the terpene *α*-farnesene was also found. Although the group of ketones was strongly represented, nonanal aldehyde was the most abundant compound with the highest mean value of relative concentration (22.0%).

**Figure 1:**
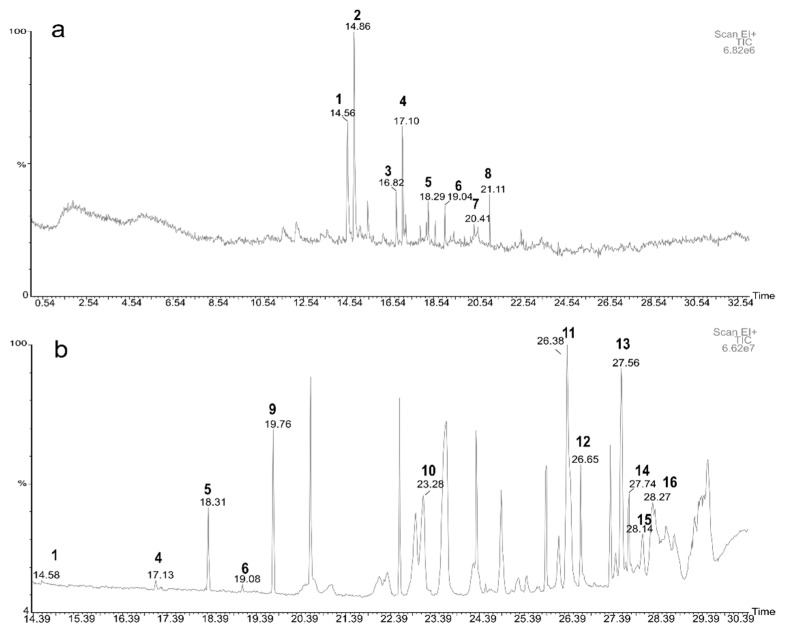
Chromatograms resulting from (**a**) HS-SPME/GC-MS and from (**b**) venom extraction/GC-MS analysis of hornets. Chemical compounds are marked by numbers (1) 2-nonanone; (2) nonanal; (3) decanal; (4) 7-nonen-2-one, 4,8-dimethyl-; (5) 2-undecanone; (6) 1,7-nonadiene, 4,8-dimethyl-; (7) geranyl acetone; (8) *α*-Farnesene; (9) tetradecane; (10) naphthalene, 1,6-dimethyl-4-(1-methylethyl)-; (11) Ethyl 9-hexadecenoate; (12) hexadecanoic acid, ethyl ester; (13) pentadecane, 8-hexyl; (14) 9-octadecenoic acid (Z)-, methyl ester; (15) 9-octadecenoic acid and (16) ethyl oleate. Scan EI + TIC: Total Ion Chromatogram in Scan ionization mode on a scale of 6.82 x 10^6^ and 6.62 x 10^7^ in (**a**) and (**b**) respectively.

Furthermore, it had the greater frequency since it was present in 100% of the individuals (Figure 1a). This compound is followed in relation to its concentration by 1,7-Nonadiene, 4,8-dimethyl- (20.4%) and the ketones: 2-nonanone (15.3%), 7-nonen-2-one, 4,8-dimethyl- (10.6%). It is important to mention the ketone 7-nonen-2-one, 4,8-dimethyl-, because it is present in more than half of the individuals and with an average value of 15.1%. In this sense, decanal aldehyde should also be highlighted as it is present in 71.4% of the samples.

### 2.2. Chemical Compounds Extracted In Vitro from the Venom Apparatus

Fourteen compounds were determined by GC-MS analysis of the venom apparatus extraction (Table 2). Most of the chemical compounds found were fatty acids and fatty acid esters (5 compounds), although ketones (3 compounds), terpenes (1 compound), and hydrocarbons such as alkanes and alkenes (2 compounds) have also been identified.

A methyl ester (9-octadecenoic acid (Z)-, methyl ester (methyl oleate)), and three ethyl esters of fatty acids (hexadecanoic acid, ethyl ester (ethyl palmitate), oleic acid ethyl ester (ethyl eoleate)) and palmitelaidic acid ethyl ester (ethyl 9-hexadecenoate) have been identified. In addition, an unsaturated fatty acid has been identified such as elaidic acid or 9-octadecenoic acid, a trans isomer of oleic acid. Three ketones were identified, these were also identified in vivo by HS-SPME/GC-MS method (2-nonanone, 2-undecanone and 7-nonen-2-one, 4,8-dimethyl-). The sesquiterpene naphthalene, 1,6-dimethyl-4-(1-methylethyl)- (cadalene) has been differentiated. Finally, one branched alkane with -hexyl as the main alkyl groups (pentadecane, 8-hexyl- (C_21_)) and an alkene with two double bonds (1,7-nonadiene, 4,8-dimethyl- (C_11:2_)) were present in the samples.

**Figure 2:**
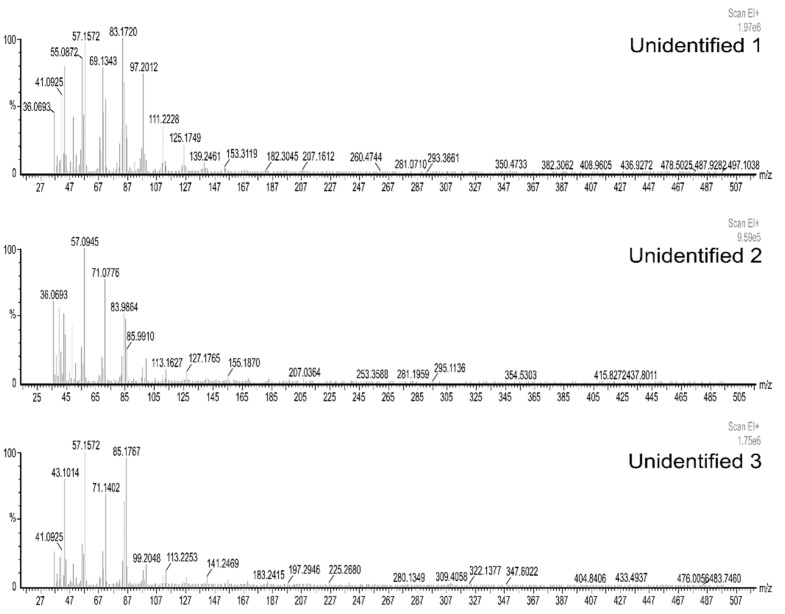
Mass spectrum of unidentified chemical compounds by in vitro extraction method. Scan EI: Scan ionization mode on a scale of 1.97 × 10^6^, 9.59 × 10^5^ and 1.75 × 10^6^ respectively.

The identification of three compounds by the NIST library and their mass spectra was not possible. However, they were considered as they were present in more than one individual. Although these compounds most likely belong to the group of alkanes and alkenes, they were named as unidentified 1, 2, and 3. The mass spectra for each of the compounds are shown in Figure 2. The unidentified 1 was present in five samples, the unidentified 2 in eight samples and the unidentified 3 in two samples. Unidentified 1 was characterized by the following major peaks 36, 41, 55, 57, 69, 83, 97, 111, and 125 (*m*/*z*), where 83 had the highest relative intensity. Unidentified 2 presented the following major peaks; 36, 57, 71, 84, 86, and 113 (*m*/*z*), highlighting 57 as the one with the highest relative intensity. Regarding the spectrum of the last compound, peaks 41, 43, 57, 71, 85, 99, and 113 (*m*/*z*) stood out, with 57 being the one with the highest relative intensity.

The alkane pentadecane, 8-hexyl- (1796.4 ng/µL) had the highest average content. This compound was also present in 53% of the individuals, being one of the most frequent (Figure 1b). Unidentified compounds 2 (424.0 ng/µL) and 1 (396.4 ng/µL) also appeared at high concentrations, followed by ethyl oleate (348.7 ng/µL). The compounds naphthalene, 1,6-dimethyl-4-(1-methylethyl)- and ethyl oleate can be highlighted as being present in five of the samples studied. On the other hand, 2-undecanone (2.6 ng/µL) and 2-nonanone (8.9 ng/µL) ketones had the lowest concentrations.

### 2.3. Chemical Compounds According to the Method of Extraction and the Caste

The compounds were analysed according to the method used and the caste of each individual. The mean values of relative concentration for each compound are shown in Table 3. For the HS-SPME/GC-MS method, individuals identified as queens had eight compounds while workers only five compounds. Nonanal; decanal; 7-nonen-2-one, 4,8-dimethyl-; geranyl acetone and *α*-farnesene were the most common chemical compounds in both castes. Queens were the only individuals that presented 2-undecanone and 2-nonanone ketones and 1,7-nonadiene alkene, 4,8- dimethyl-, the latter being the one with the highest concentration (20.4% relative). The workers according to the HS-SPME/GC-MS method did not present any exclusive compound. Nonanal was by far the major compound in terms of relative concentration in both castes. Although no significant differences were found between the mean content as a function of caste, nonanal (22.9%) and 7-nonen-2-one, 4,8-dimethyl- (13,7%) presented slightly higher values in the individuals classified as queens. Regarding workers, decanal (4.7%) and *α*-farnesene (3.1%) showed higher average values.

In the case of the venom extraction method, the workers presented the greatest diversity of compounds (11 compounds), 2 of them in common with the queen samples (pentadecane, 8-hexyl- and naphthalene, 1,6-dimethyl-4-(1-methylethyl)-). The content of these two compounds was slightly higher in the workers. The most abundant compound in the workers was pentadecane, 8-hexyl- (2122.9 ng/µL) followed by ethyl oleate (348.7 ng/µL).

A Kruskal-Wallis analysis was carried out. The analysis allowed to observe the differences between the medians of the concentrations of the chemical compounds present in both workers and queens. The concentrations were compared for each method used for the extraction of the compounds. The compounds derived from the extraction of the venom apparatus; naphthalene, 1,6-dimethyl-4- (1-methylethyl)- and pentadecane, 8-hexyl- were present both in workers and the founders. The Kruskal-Wallis test calculated the average rank for pentadecane, 8-hexyl- (3.8 workers and 2.0 founders). Given that the *p*-value was greater than 0.05, there was no statistically significant difference between the medians at the level of confidence of 95.0%. As for naphthalene, 1,6-dimethyl-4- (1-methylethyl)-, the same occurred, with an average rank of 4.0 in workers and 1.5 in founders.

In the case of chemical compounds detected by HS-SPME/GC-MS; 7-nonen-2-one, 4,8-dimethyl- presented an average rank for workers of 1.0 while 3.0 for founders. With a *p*-value = 0.2 did not present significant differences. The same occurred for the decanal content between workers and founders (average rank 3.3 for workers and 2.5 for founders; *p*-value = 0.6). Since the *p*-value is greater than or equal to 0.05, (*p*-value = 1.0) there was no statistically significant difference between the nonanal content between founders (Average Rank 4.0) and workers (average rank 4.0). Even though *α*-famesene and geranyl acetone appear both in queens and workers, the number of samples for each caste did not allow the Kruskal-Wallis test. Based on this, the main differences are attributed to the presence/absence of certain compounds already detailed in previous paragraphs.

## 3. Discussion

The use of both procedures could ensure a complementary identification of volatile and semi-volatile chemical compounds that could participate in the intra and interspecific interaction of *V. velutina*. Headspace solid-phase microextraction (HS-SPME) is an effective, fast, and easy-to-use technique in the identification of semiochemicals of *V. velutina* [7,8]. Some studies have used this technique to identify alarm compounds, sex pheromones or other volatile chemical compounds from the venom gland or the cuticle of *V. velutina* [4,6,7,8]. The venom gland extraction is another method that has also been carried out in *V. velutina* [7,8]. Venom gland is the source of the alarm pheromone [7], although it can also produce sex pheromone in several polistine wasp species [6].

Table 3 includes information supporting the role as semiochemicals of the compounds determined in this study. Nonanal was the most abundant and frequent compound identified in hornet individuals by HS-SPME/GC-MS. It is a saturated fatty aldehyde with multiple functions in the communication of species of wasps and hornets. Couto et al. [14]. identified nonanal as a volatile aliphatic odorant using the single sensillum extracellular recording (SSR) technique in *V. velutina*. Furthermore, olfactory learning and long-term olfactory memory in the Asian hornet were tested using gynes of *V. velutina,* which responded significantly to nonanal [16]. This olfactory response to nonanal has also been studied in the relationships of *Apis cerana* with the Asian hornet [17]. Nonanal was identified in alarm pheromones of social wasps as *Polybia* and as a cuticular aldehyde attractive for females of *Cotesia* wasps [18,19]. It is considered a key signal volatile of tobacco plants to attract female moths to oviposit [20]. Decanal have been detected coming from the venom gland in Vespidae such as *Polybioides raphigastra* [21]. This compound together with nonanal has been mentioned as part of the swarming trail pheromone secreted by Richard’s gland in *P. sericea* [22]. Different alkanes, methyl-, hexyl-, and ethyl-branched alkanes, and alkenes have been studied as chemical compounds with a role in insect communication. These compounds were mostly considered cuticular hydrocarbons (CHCs), acting during interactions with nestmates [3,4,5,6,7,8,10,11,14] or with other species [23]. Alkanes act as cues in social insects but vary intraspecifically according to factors such as the stage of development, genetics, or temperature [24]. In honeybees, nonadecane (C19) was closely related to an alarm signal secreted by the mandibular gland [25]. Heneicosane (C_21_) was identified as a cuticular hydrocarbon in both *V. velutina* [3] and *V. crabro* [26] and as a volatile defence in the hive environment [27]. Heptacosane (C_27_) has been studied in *V. velutina* as a semiochemical recognized by sensory neurons within the basiconic sensilla of the hornet [16]. This compound and nonadecane (C_19_), are recognized by *V. velutina* when it is released by *S. tuberosa* for the dispersal of its seeds [23]. Octacosane (C_28_) is another cuticular hydrocarbon found in *V. velutina* [3]. Hentriacontane (C_31_) has been identified in *V. velutina* [3]. Regarding branched alkanes, the diversity of the group makes their presence more specific in certain species. Some of these compounds have been released by plants and recognized by insects as a sign of plant-insect interaction, thus pentadecane, 8-hexyl-(C_21_) appears on the surface of sorghum seedlings as a mechanism of resistance to the shoot fly [28]. Alkenes are a group of compounds widely reported as cuticular hydrocarbons, although they can also behave as pheromones [29]. In this study 1,7-nonadiene, 4,8-dimethyl- were identified. This was detected as a volatile compound from fractions of kaffir lime leaves (*Citrus Hystrix*) [30].

Regarding *V. velutina*, the ketones 2-nonanone and 7-nonen-2-one, 4,8-dimethyl- were mentioned as alarm pheromones [7,8]. Another ketone identified in *V. velutina* was 2-undecanone. This has been studied as a female volatile isolated from the venom gland [4]. The ketones 2-nonanone and 2-undecanone are also mentioned as volatile compounds in the study of alarm pheromone in the venom of *Polybia occidentalis* and *P. sericea* [18], in *V. orientalis* [31] and as a pheromone for the alert nest mates in *Dolichovespula maculata* [32]. Another of the ketones identified in the venom gland was geranyl acetone, a monoterpene ketone also referenced as a volatile compound in the venom of *Polistes dominulus*, *P. gallicus*, *P. nimphus*, *P. sulcifer,* and *P. olivaceus* [33,34] and from the venom sac of four *Ropalidia* species [35]. As in this study, this last chemical compound has also been identified in both workers and founders of *P. dominulus* [36].

Terpene compounds are well known in nature among the structural types of pheromones. HS-SPME is a technique known for the extraction of these types of compounds [37]. In this study it allowed the identification of α-farnesene. The sesquiterpene farnesene most likely functions as both alarm pheromones and defensive products in social insects [38]. This sesquiterpene is a volatile compound that attracts the social wasp *Polybia fastidiosuscula* to corn when it is attacked by the herbivory of *Spodoptera frugiperda* [39]. In addition, other terpenes found were naphthalene, 1,6-dimethyl-4-(1-methylethyl)- (cadalene) identified for the first time in *V. velutina*. The latter is a sesquiterpene with repellent properties used by some plants such as *Teucrium polium* subsp. capitatum (L.) [40].

The compounds belonging to the group of fatty acid and fatty acid esters have been investigated as attractive compounds for *V. velutina* [15,41]. They are also part of the pheromone composition of various species of insects, including social wasps. Ethyl oleate, an important primer pheromone already studied in the honeybee [42], has also been found as a component of the venom sac of three species of stenogastrine wasps (*Eustenogaster fraternal*, *Liostenogaster flavolineta,* and *Parischnogaster mellyi*) [43]. 9-Octadecenoic acid (Z)-, methyl ester (methyl oleate) has been studied as a chemical compound involved in the recognition of nestmate being secreted by Dufour’s gland in *Polistes* wasps [44]. In insects such as hornets and honeybees, hexadecanoic acid (ethyl palmitate) is part of the brood pheromone, and it inhibits the development of the ovary to stimulate brood care. In addition, the release of this compound by the queen has been demonstrated, thus it also is part of the queen pheromone blend [45]. Ethyl 9-hexadecenoate (ethyl 9-hexadecenoate) has been considered as a sex pheromone in some Hymenoptera families such as Eurytomidae [46] and Braconidae [47]. Finally, 9-Octadecenoic acid (oleic acid) has been studied as a chemical component derived from the glands of Hymenoptera as from the mandible glands of *V. crabro* [48] or as chemical secretion of the sternal glands of *Polistes* social wasps [49].

**Table 3:** Chemical compounds depending on both methods and according to the caste of each individual.

Chemical Compounds	Method				Biological Function
	In Vitro (ng/µL)		In Vivo (%)		
	Queen	Worker	Queen	Worker	
**Aldehydes (13.3%)**					
Nonanal	-	-	22.9	20.7	Volatile aliphatic odorants [10]
					Alarm pheromone [50]
					Trail pheromone [22]
					Sex pheromone [16]
Decanal	-	-	3.8	4.7	Trail pheromone [22]
**Branched alkanes (6.7%)**					
Pentadecane, 8-hexyl-	164.0	2122.9	-	-	Allelomone [28]
**Alkenes (6.7%)**					
1,7-Nonadiene, 4,8-dimethyl-	-	9.0	20.4	-	
**Ketones (26.7%)**					
2-Nonanone	-	8.9	15.3	-	Alarm pheromone [7,8]
2-Undecanone	-	2.6	4.3		Alarm pheromone [7,8]
7-Nonen-2-one, 4,8-dimethyl-	-	12.8	13.7	1.0	Alarm pheromone [7,8,50]
Geranyl acetone			2.7	2.7	Venom sac volatile compound [33,34,35]
**Terpenes (13.3%)**					
Naphthalene, 1,6-dimethyl-4-(1-methylethyl)-	10.6	16.8	-	-	
*α*-Farnesene			2.5	3.1	Alarm pheromones [38]
					Allelomone [39]
**Fatty acid and fatty acid esters (33.3%)**					
Ethyl oleate	-	348.7			Primer pheromone [42]
					Component of the venom sac [43]
9-Octadecenoic acid (Z)-, methyl ester	-	37.0			Recognition of nestmate [44]
9-Octadecenoic acid	-	84.8			Glandular chemical component [48,49]
Ethyl 9-hexadecenoate	-	30.3			Sex pheromone [46,47]
Hexadecanoic acid, ethyl ester	-	35.5			Queen pheromone [45]

() Parentheses specify the relative frequency.

## 4. Materials and Methods

### 4.1. Procedure to Capture V. velutina Specimens and Caste Identification

A total of 43 individuals of *V. velutina* were captured during the Spring–Autumn period of 2018 in Galicia. Individuals were classified as 4 queens and 39 workers, based on the description done by Monceau et al. [13]. The identification of the queens was also confirmed due to their collection in embryonic nests. For the in vivo experimentation, 7 hornets were kept alive. After this, the 43 hornets were frozen at −80 °C for in vitro analysis.

### 4.2. Collection of VOCs Released “In Vivo” by Headspace-Solid Phase Microextraction (HS-SPME)

**Figure 3:**
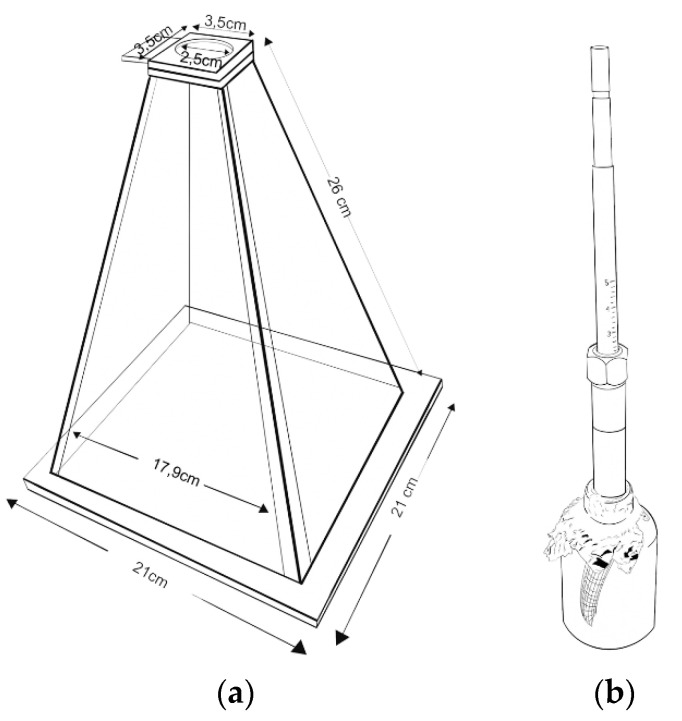
Experimental design for the in vivo analysis using HS-SPME/GC-MS method. (**a**) Pyramid acrylic box and (**b**) design of the glass container in which volatiles are collected by SPME fiber.

Seven hornets (4 queens and 3 workers) were kept one by one the same day of their capture in a pyramidal acrylic box (Figure 3a). The structure of this pyramid is useful to introduce the hornets to a glass vial. The pyramid was also darkened, except for its vertex, by placing a black cloth on it. Thereby, the hornets tended to exit the pyramid through the vertex. Each hornet was then transferred to a glass vial with aluminium foil placed on both sides of the container to limit air movement (Figure 3b). A 65 µm thick polydimethylsiloxane/divinylbenzene (PDMS/DVB) fibre (Supelco SPME fibre 57326U, Darmstadt, Germany) was fixed at the glass bottle, with the hollow SPME needle stuck through the aluminium foil, exposing the fibre, close to the hornet, to adsorb the volatile compounds present in the headspace. Before use, the SPME fibre was preconditioned and thermally cleaned. This was done thermally by exposing the fibre at a conditioning temperature of 250 °C for 30 min in the GC injection port. The chosen extraction mode was Headspace (HS) since the analytes of interest are highly volatile. Thus, the fibre was introduced into the glass vial with the hornet and exposed in dark for 30 min at 25 °C. After this period, the fibre was retracted and transferred to the gas phase chromatograph (GC) injector where the compounds were desorbed for 5 min.

### 4.3. Removal of the Sting Apparatus of Hornets and “In Vitro” Extraction of Chemical Compounds

All specimens of *V. velutina* were used for sting apparatus extraction. Extraction of the sting apparatus is an adaptation of the method used by Hunt et al. [50]. Hornet individuals were pooled. Thirteen pools of 3 sting apparatus (13 × 3) were made for the workers and 2 pools of 2 sting apparatus (2 × 2) for the queens, obtaining a total of 15 samples. Prior and during the extraction, the samples were kept on ice. The stingers were extracted with 1 mL of methylene chloride and 100 µL of *n*-tetradecane (50 ng/mL), which has been used as an internal standard. Sting apparatus were crushed with a glass rod for 2 min and centrifuged (4000 rpm for 20 min at 4 °C). The supernatant was collected and 1 μL of this solution was injected into the gas chromatography-mass spectrometer.

### 4.4. Gas Chromatography-Mass Spectrometry (GC-MS) Procedure

Gas chromatographic analyses were performed using a Perkin Elmer system with a Clarus^®^ 580 GC module and a Clarus^®^ SQ 8 S MS module, equipped with DB-5MS fused-silica column (30 m/0.25 mm i.d., film thickness 0.25 μm; J & W Scientific, Inc., Folsom, CA, USA). The injection was done in splitless mode, and the fibre desorption was carried out for 5 min at 250 °C. The oven temperature was set at 40 °C and held isothermal for 5 min, then programmed from 40 °C to 75 °C (5 °C/min) and from 75 °C to 230 °C (10 °C/min), then maintained at 230 °C for 5 min. Helium was used as carrier gas at a constant velocity of 1.45 mL/min. The mass spectrum was obtained from an ionization energy of 70 eV. The transfer line and the ionization source temperatures were 250 °C and 230 °C, respectively. The software Turbomass (software version 6.1.0, Perkin Elmer, Shelton, CT, USA) for Windows was used for data acquisition. The identity of the components was assigned by comparison of their retention indices, relative to C7-C40 n-alkane indices and GC-MS spectra from a commercial MS database (NIST 2011 mass spectral library). Compounds were quantified as area percentages of total volatiles using the relative values directly obtained from peak total ion current (TIC). Quantification of the identified compounds was done based on the internal standard method for the sting apparatus extractions and through the area percentages of total volatiles using the relative values directly obtained from peak total ion current (TIC) for the HS-SPME/GC-MS extractions.

### 4.5. Statistical Analysis

Relative concentrations calculated as a function of area (%) were analysed statistically with IBM SPSS^®^ Statistics 24 and XLSTAT software version 2012.04.1. A non-parametric Kruskal-Wallis analysis was performed to differentiate the concentrations between the chemical compounds present in both workers and foundresses for each method.

## 5. Conclusions

Fifteen chemical compounds have been reported. Some of the compounds have been previously confirmed in this hornet, such as 2-nonanone ketones; 2-undecanone and 7-nonen-2-one, 4,8-dimethyl-. Other compounds detected in this study were detected for the first time, as well as other ketones, aldehydes, alkanes and alkenes, terpenes, and fatty acid and fatty acid esters. The use of both “in vivo” and “in vitro” determination methods by HS-SPME/GC-MS, allowed increasing our knowledge about the chemical composition released by *V. velutina*. Furthermore, this information is a necessary step in the study of the behaviour of this species.

## Figures and Tables

**Table 1:** Volatile compounds identified by HS-SPME/GC-MS.

Chemical Compounds	N (%)	RT	Mean%	SD	Min	Max	^a^ LRI	^b^ LRI	ID
2-Nonanone	42.9	14.5	15.3	11.0	4.4	26.3	1088	1090	RI, MS
Nonanal	100.0	14.9	22.0	16.6	5.4	48.3	1099	1098	RI, MS
Decanal	71.4	16.8	4.4	3.6	1.4	9.8	1199	1200	RI, MS
7-Nonen-2-one, 4,8-dimethyl-	57.1	17.1	10.6	9.5	1.0	23.8	1217	1227 *	RI, MS
2-Undecanone	14.3	18.2	4.3		4.3	4.3	1274	1273	RI, MS
1,7-Nonadiene, 4,8-dimethyl-	14.3	19.1	20.4		20.4	20.4	1343		MS
Geranyl acetone	28.6	20.4	2.7	0.0	2.7	2.7	1438	1432	RI, MS
α-Farnesene	28.6	21.1	2.8	0.5	2.5	3.1	1491	1494	RI, MS

N (%): Percentage of the number of samples in which the compound is present; RT: Retention time; Mean%: Relative concentration of components in area percentage; SD: Standard Deviation; ^a^ LRI: linear retention index determined on a DB-5 MS fused silica column relative to a series of n-alkanes (C_7_–C_40_); ^b^ LRI: Linear retention index theoretical obtained through the NIST Chemistry Web Book, SRD 69; *: Linear retention index theoretical obtained from Thiéry et al. [8], and ID: Resources by which the compound was identified.

**Table 2:** Main compounds detected by venom apparatus extraction.

Chemical Compounds	N(%)	RT	ng/µL	SD	Min	Max	^a^ LRI	^b^ LRI	ID
2-Nonanone	6.7	14.6	8.9		8.9	8.9	1090	1090	RI, MS
7-Nonen-2-one, 4,8-dimethyl-	20.0	17.1	12.8	7.1	8.0	20.9	1218	1227 *	RI, MS
2-Undecanone	6.7	18.2	2.6		2.6	2.6	1286	1273	RI, MS
1,7-Nonadiene, 4,8-dimethyl-	13.3	19.1	9.0	6.0	4.8	13.2	1343		MS
**Tetradecane (Standard)**	100.0	19.8	50.0	0.0	50.0	50.0	1391	1400	RI, MS
Naphthalene, 1,6-dimethyl-4-(1-methylethyl)-	33.3	23.3	14.3	4.2	8.4	19.5	1670	1672	RI, MS
Unidentified 1	33.3	24.9	396.4	331.5	69.3	805.7	1814		
Unidentified 2	53.3	26.1	424.0	1000.8	23.8	2899.3	1930		
Ethyl 9-hexadecenoate	13.3	26.4	30.3	9.3	23.8	36.9	1958	1978.3	RI, MS
Hexadecanoic acid, ethyl ester	20.0	26.6	35.5	29.0	4.7	62.3	1982	1993	RI, MS
Pentadecane, 8-hexyl-	40.0	27.5	1796.4	3561.1	141.2	9054.8	2067	2045	RI, MS
9-Octadecenoic acid (Z)-, methyl ester	13.3	27.7	37.0	0.9	36.4	37.7	2091	2103	RI, MS
Unidentified 3	13.3	27.8	39.9	0.0	39.9	39.9	2102		
9-octadecenoic acid	13.3	28.2	84.8	78.2	29.5	140.1	2138	2141	RI, MS
Ethyl oleate	33.3	28.3	348.7	546.8	40.4	1319.6	2150	2168.8	RI, MS

N (%): Percentage of the number of samples in which the compound is present ng/µL: Concentration according to standard; RT: retention time; SD: standard deviation; ^a^ LRI, linear retention index determined on a DB-5 MS fused silica column relative to a series of n-alkanes (C_7_–C_40_); ^b^ LRI: linear retention index theoretical obtained through the NIST Chemistry Web Book, SRD 69; *: Linear retention index theoretical obtained from Thiéry et al. [8], and ID: Resources by which the compound was identified.

## Data Availability

The datasets generated during and/or analyzed during the current study are available from the corresponding author on reasonable request.
